# Supplementary material for: Carafe enables high quality in silico spectral library generation for data-independent acquisition proteomics
Source: Nat Commun. 2025 Nov 6;16:9815. doi: 10.1038/s41467-025-64928-4 (PMC12592563; doi:10.1038/s41467-025-64928-4)
Supplement: Supplementary file 2 — Description of Additional Supplementary Files [file 41467_2025_64928_MOESM2_ESM.pdf]

Description of Additional Supplementary Files

File Name: Supplementary Data 1

Description: LC and MS parameter settings.

File Name: Supplementary Data 2

Description: The MS/MS files used in the study.
