# Supplementary material for: Carafe enables high quality in silico spectral library generation for data-independent acquisition proteomics
Source: Nat Commun. 2025 Nov 6;16:9815. doi: 10.1038/s41467-025-64928-4 (PMC12592563; doi:10.1038/s41467-025-64928-4)
Supplement: Supplementary file 5 — Reporting Summary [file 41467_2025_64928_MOESM5_ESM.pdf]

nature portfolio

Corresponding author(s): Michael J. MacCoss, William S. Noble

Last updated by author(s): Sep 11, 2025

Reporting Summary

Nature Portfolio wishes to improve the reproducibility of the work that we publish. This form provides structure for consistency and transparency in reporting. For further information on Nature Portfolio policies, see our Editorial Policies and the Editorial Policy Checklist.

Statistics

For all statistical analyses, confirm that the following items are present in the figure legend, table legend, main text, or Methods section.

n/a	Confirmed	
☐	☒	The exact sample size (*n*) for each experimental group/condition, given as a discrete number and unit of measurement
☐	☒	A statement on whether measurements were taken from distinct samples or whether the same sample was measured repeatedly
☐	☒	The statistical test(s) used AND whether they are one- or two-sided<br>*Only common tests should be described solely by name; describe more complex techniques in the Methods section.*
☒	☐	A description of all covariates tested
☒	☐	A description of any assumptions or corrections, such as tests of normality and adjustment for multiple comparisons
☐	☒	A full description of the statistical parameters including central tendency (e.g. means) or other basic estimates (e.g. regression coefficient) AND variation (e.g. standard deviation) or associated estimates of uncertainty (e.g. confidence intervals)
☐	☒	For null hypothesis testing, the test statistic (e.g. *F*, *t*, *r*) with confidence intervals, effect sizes, degrees of freedom and *P* value noted<br>*Give P values as exact values whenever suitable.*
☒	☐	For Bayesian analysis, information on the choice of priors and Markov chain Monte Carlo settings
☒	☐	For hierarchical and complex designs, identification of the appropriate level for tests and full reporting of outcomes
☐	☒	Estimates of effect sizes (e.g. Cohen's *d*, Pearson's *r*), indicating how they were calculated

Our web collection on statistics for biologists contains articles on many of the points above.

Software and code

Policy information about availability of computer code

Data collection	Mass spectrometry datasets generated in this study were acquired using the Thermo Astral, the Thermo Lumos Fusion Tribrid, and the Thermo Exploris 480 mass spectrometers. No custom software was used to collect the data in the study.
Data analysis	Data in this study was processed and analyzed using the following software tools:<br>- ProteoWizard (version 3.0.24031)<br>- DIA-NN (version 1.8.1)<br>- EncyclopeDIA (version 2.12.30)<br>- Oktoberfest (version 0.6.2)<br>- nf-skyline-dia-ms (revision: ef37e6e3cf)<br>- FDRBench (version 0.0.1)<br>- R (version 4.3.1)<br>- R package impute (version 1.76.0)<br>Additionally, Carafe's source code is available under the Apache 2.0 license at https://github.com/Noble-Lab/Carafe. The Nextflow workflow for Carafe is available under the Apache 2.0 license at https://nf-carafe-ai-ms.readthedocs.io/. The Skyline version with Carafe integrated is available at https://proteome.gs.washington.edu/~dshteyn/SkylineCarafePreview/. The source code of the customized AlphaPeptDeep used in this study, with peak masking support, is available under the Apache 2.0 license at https://github.com/wenbostar/alphapeptdeep_dia.

For manuscripts utilizing custom algorithms or software that are central to the research but not yet described in published literature, software must be made available to editors and reviewers. We strongly encourage code deposition in a community repository (e.g. GitHub). See the Nature Portfolio guidelines for submitting code & software for further information.

Data

Policy information about availability of data

All manuscripts must include a data availability statement. This statement should provide the following information, where applicable:

- Accession codes, unique identifiers, or web links for publicly available datasets
- A description of any restrictions on data availability
- For clinical datasets or third party data, please ensure that the statement adheres to our policy

The MS/MS datasets generated in this study have been deposited to Panorama Public (ProteomeXchangeidentifier: PXD056793) and are available at https://panoramaweb.org/Carafe.url. The Q Exactive HF-X dataset and the TripleTOF 5600 dataset were downloaded from PRIDE with accession number PXD028735. The LFQBench dataset was downloaded from PRIDE with accession number PXD002952. Both the yeast phosphoproteome DIA dataset with spike-in synthetic phosphopeptides and the EGF-stimulated HeLa phosphoproteome DIA dataset were downloaded from MassIVE with accession number MSV000093613.

Research involving human participants, their data, or biological material

Policy information about studies with human participants or human data. See also policy information about sex, gender (identity/presentation), and sexual orientation and race, ethnicity and racism.

Reporting on sex and gender	Not applicable
Reporting on race, ethnicity, or other socially relevant groupings	Not applicable
Population characteristics	Not applicable
Recruitment	Not applicable
Ethics oversight	Not applicable

Note that full information on the approval of the study protocol must also be provided in the manuscript.

Field-specific reporting

Please select the one below that is the best fit for your research. If you are not sure, read the appropriate sections before making your selection.

[x] Life sciences [ ] Behavioural & social sciences [ ] Ecological, evolutionary & environmental sciences

For a reference copy of the document with all sections, see nature.com/documents/nr-reporting-summary-flat.pdf

Life sciences study design

All studies must disclose on these points even when the disclosure is negative.

Sample size	For datasets generated in this study, each dataset includes MS runs from a human cell line sample and a yeast sample or a metaproteome sample. For datasets downloaded from public databases, the sample sizes were determined by the authors of the original studies.
Data exclusions	For model training, only one replicate MS run was used for each dataset. For testing, one or more MS runs were used.
Replication	All attempts to replicate the improvement in peptide detection using fine-tuned libraries were successful.
Randomization	Randomization is not relevant because the datasets generated in this study include no separate experimental groups for statistical comparison.
Blinding	Blinding is not relevant because the study focuses on software development.

Reporting for specific materials, systems and methods

We require information from authors about some types of materials, experimental systems and methods used in many studies. Here, indicate whether each material, system or method listed is relevant to your study. If you are not sure if a list item applies to your research, read the appropriate section before selecting a response.

Materials & experimental systems

n/a	Involved in the study	
☒	☐	Antibodies
☐	☒	Eukaryotic cell lines
☒	☐	Palaeontology and archaeology
☒	☐	Animals and other organisms
☒	☐	Clinical data
☒	☐	Dual use research of concern
☒	☐	Plants

Methods

n/a	Involved in the study	
☒	☐	ChIP-seq
☒	☐	Flow cytometry
☒	☐	MRI-based neuroimaging

Eukaryotic cell lines

Policy information about cell lines and Sex and Gender in Research

Cell line source(s)	HeLa S3 cell lines and the S288C S. cerevisiae strain
Authentication	The cell lines used were not autenticated
Mycoplasma contamination	The HeLa S3 cell lines used for global proteome were not tested for mycoplasma contamination. The HeLa S3 cell lines used for phosphoproteome were tested for mycoplasma contamination.
Commonly misidentified lines (See ICLAC register)	N/A

Plants

Seed stocks	N/A
Novel plant genotypes	N/A
Authentication	N/A
